# Educational Impact of a 3D Canine Vascular Simulator for Learning Anatomy and Interventional Radiology Techniques in Veterinary Training

**DOI:** 10.3390/vetsci12121139

**Published:** 2025-11-29

**Authors:** Sandra Lopez-Minguez, Iris Urbano, Ignacio de Blas, Cantal del Rio-Martinez, Cristina Bonastre, Jose Andres Guirola, Jose Benito Rodriguez, Francisco Javier Miana-Mena, Carolina Serrano-Casorran

**Affiliations:** 1 Faculty of Veterinary Medicine, Universidad de Zaragoza, 50013 Zaragoza, Spain; sanlomin@unizar.es (S.L.-M.); 805866@unizar.es (I.U.); ndeblas@unizar.es (I.d.B.); cbonastr@unizar.es (C.B.); jrodgom@unizar.es (J.B.R.); jmiana@unizar.es (F.J.M.-M.); carolse@unizar.es (C.S.-C.); 2 Faculty of Medicine, Universidad de Zaragoza, 50009 Zaragoza, Spain; joseandresguirola@gmail.com

**Keywords:** veterinary education, simulation-based learning, education techniques, surgery simulator, interventional radiology

## Abstract

Background: Simulation-based teaching is increasingly important in veterinary training, especially for learning anatomy and interventional techniques where real-life exposure is limited. Methods: A handcrafted 3D simulator replicating the canine abdominal arterial system was developed and tested with 80 veterinary students and graduates. A quasi-experimental pre–post design evaluated theoretical knowledge, practical skills, and self-perceived competence after a structured training session including anatomical identification, catheter navigation, and the Seldinger technique. Results: Post-training results showed significant improvements in all domains. Theoretical test scores increased by over 25% (*p* < 0.001), and more than 85% of participants correctly identified target arteries after training. Navigation success rose to 90%, with shorter execution times and fewer errors. Participants reported increased confidence and satisfaction with the realism and educational value of the simulator. Conclusions: The 3D vascular simulator effectively enhanced learning outcomes and confidence in interventional radiology. Its low-cost, handcrafted design makes it a feasible and valuable educational tool for veterinary institutions lacking access to high-fidelity commercial simulators.

## Simple Summary

Understanding the anatomy of the canine vascular system and performing interventional procedures are complex skills for veterinary students to acquire. Traditional teaching methods often provide limited opportunities for hands-on practice due to ethical, technical, and logistical constraints. To address this challenge, we developed a low-cost, handcrafted simulator that reproduces the main abdominal arteries of the dog. The simulator allows students to practice catheter navigation and vascular access techniques safely, without the need for live animals. Our study evaluated its educational impact among veterinary students and graduates at the University of Zaragoza. After a short training session, participants significantly improved their knowledge of vascular anatomy, manual dexterity, and confidence in performing interventional techniques. They also expressed high satisfaction with the learning experience, highlighting the realism and clarity of the simulator. These results show that simple, affordable simulation tools can effectively complement traditional teaching, making complex anatomical concepts easier to understand and promoting early exposure to interventional radiology in veterinary education.

## 1. Introduction

Teaching anatomy and minimally invasive procedures represents one of the most demanding challenges in veterinary education. Anatomical structures, particularly those of the vascular system, have a complex three-dimensional organization that is difficult to understand through traditional lecture-based methods. At the same time, developing practical competences requires technical accuracy, spatial reasoning, and refined coordination, which students cannot always achieve in real clinical settings due to ethical, logistical, or financial constraints [1,2].

In response to these limitations, simulation-based education has emerged as an effective and flexible strategy in health sciences training. As Okuda et al. (2009) described, simulation enables the safe and standardized replication of clinical scenarios, allowing learners to practice repeatedly, receive immediate feedback, and develop both technical skills and clinical judgment without compromising patient safety. These features align with adult learning principles and have led to the widespread adoption of simulation in both human and veterinary education [3]. A growing body of research supports the idea that simulation-based training enhances technical skills, deepens anatomical understanding, improves learners’ confidence, and facilitates objective assessment of their progress [4,5,6]. In veterinary medicine, simulators have been developed for various procedures such as laparoscopy, rectalis palpation, or regional anesthesia, in both physical and digital formats, including virtual and augmented reality [4,6,7,8]. However, most of these models are adaptations from human medicine, with limited tailoring to specific animal anatomy and often lacking educational validation [1,4,9].

In particular, simulation tools designed for studying the vascular system of small animals remain scarce, despite their relevance for advanced clinical procedures [1,3]. Our review of the existing literature revealed no published training models replicating the abdominal arterial system of the dog, exposing a significant gap in veterinary education. Allowing students to explore, identify, and perform interventions on a simulated vascular system would not only improve anatomical learning but also offer an accessible introduction to techniques such as endovascular navigation and selective catheterization, which are essential skills in interventional radiology.

Interventional radiology (IR) is a field of medicine that uses image-guided access through natural or vascular pathways to reach target organs for diagnostic or therapeutic purposes. Mastery of IR requires in-depth anatomical knowledge and the ability to work with specialized instruments such as guidewires, catheters, and stents [10,11]. While IR is an established subspecialty in human medicine, training opportunities for residents remain limited. A recent international survey conducted by Makris et al. (2020) revealed that a large proportion of medical trainees reported inadequate access to vascular procedure training. Moreover, many indicated that earlier exposure to IR might have positively influenced their career decisions. These findings underscore the significance of providing structured simulation-based learning experiences at early stages of clinical education, even more so in veterinary medicine, where interventional radiology is still emerging as a field and often remains unknown to students until late in their careers. In 2005, Chick Weisse founded the first university veterinary IR service at the Ryan Veterinary Hospital (University of Pennsylvania), helping to define the field through clinical innovation and translational collaboration [12,13].

Despite growing clinical interest, IR remains absent from undergraduate veterinary curricula. This lack of structured teaching limits students’ exposure to interventional concepts and procedures, while the inherent complexity of the vascular system continues to pose a major educational challenge, particularly in the absence of resources that promote spatial understanding through hands-on experience.

Simulation-based education is also consistent with cognitive load theory and the principles of deliberate practice, which emphasize the importance of structured, repetitive training in controlled environments for efficient skill acquisition and long-term retention.

Therefore, the present study describes the design, development, and assessment of a low-cost, handcrafted simulator that faithfully reproduces the dog’s abdominal arterial system, both anatomically and functionally. Specifically, we aimed to evaluate whether a single structured training session improved theoretical knowledge of vascular anatomy, assess changes in practical skills related to catheter navigation and the Seldinger technique, and explore the effect on learners’ self-perceived competence and confidence.

## 2. Materials and Methods

### 2.1. Experimental Design

The experimental phase was performed in the skills laboratory of the Faculty of Veterinary Medicine at the Universidad de Zaragoza (Spain). Participants were divided into two groups: Group A consisted of undergraduate veterinary students, while Group B included veterinary graduates with diverse specializations. The “graduates” group included interns and faculty members from the Veterinary Faculty of the University of Zaragoza. Their professional experience varied widely; some were clinicians (>1 year of experience), while others were involved in teaching or research (Anatomy and physiology Department). All participants underwent the same procedures in the same sequence, without prior information about the topics covered or the nature of the tasks they would face.

Due to the length of the evaluation process, participants were scheduled on different days and time slots randomly assigned and independent of group membership to avoid potential bias. Sessions were conducted both in the morning and afternoon. Each session lasted approximately 50 min and included a theoretical assessment, a practical task, a structured teaching session, and a final round of theoretical and practical evaluations. The pre- and post-intervention assessments were designed to be equivalent in difficulty level, though the questions and practical challenges varied in content.

### 2.2. Simulator Design and Construction

Two simulators replicating the canine abdominal arterial system were employed. They were built using transparent PVC tubing of various diameters to simulate the truncus coeliacus abdominalis and its main branches, sealed at the junctions to permit intraluminal navigation.

**Figure 1:**
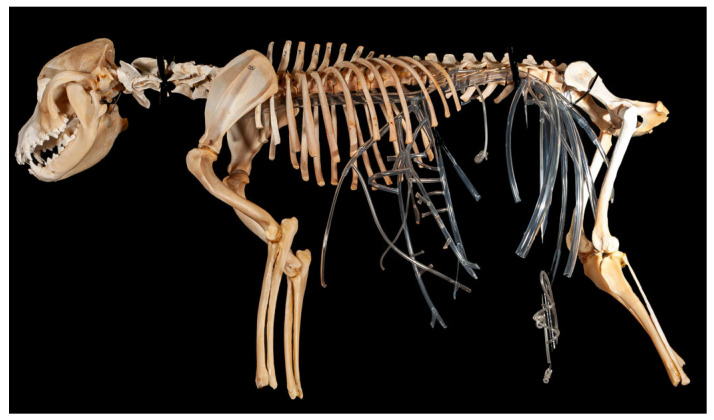
Simulator 1.

Simulator 1 was mounted onto a canine skeleton to provide osteological references, essential in interventional radiology (Figure 1). The bones were obtained by boiling the cadaver in water and then whitening them in a water–hydrogen peroxide solution. Once cleaned and dried, the bones were assembled with silicone, and the vertebral column was stabilized by inserting a rigid tube through the medullary canal to maintain alignment. Using this framework, the main aortic branches were positioned according to vertebral landmarks: celiac trunk, cranial mesenteric, renal, caudal mesenteric, iliac, and sacral arteries.

**Figure 2:**
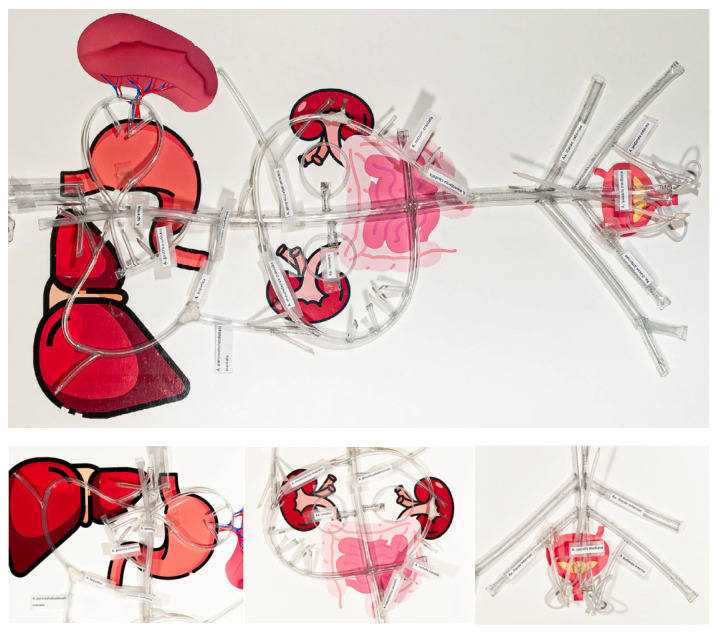
Simulator 2, with anatomical references.

Simulator 2 was a simplified model designed for endovascular navigation. It was placed in sternal recumbency over a printed diagram of the canine abdominal organs, which provided anatomical reference points for the correct positioning of the vessels (Figure 2). An introducer was pre-inserted into the femoral artery to facilitate the use of guidewires and catheters.

For both simulators, the arterial system was reconstructed with flexible PVC tubes ranging from 4 to 30 mm in internal diameter. In Simulator 2, tube diameters were intentionally oversized to facilitate catheter and guidewire movement. All connections were bonded and sealed using a cyanoacrylate adhesive (Super Glue type) suitable for PVC. At each junction, an opening was created in the larger tube with the same internal diameter as the smaller tube being connected, allowing external adhesion of the smaller tube’s edge to the larger one and preventing internal ridges to improve navigability.

**Figure 3:**
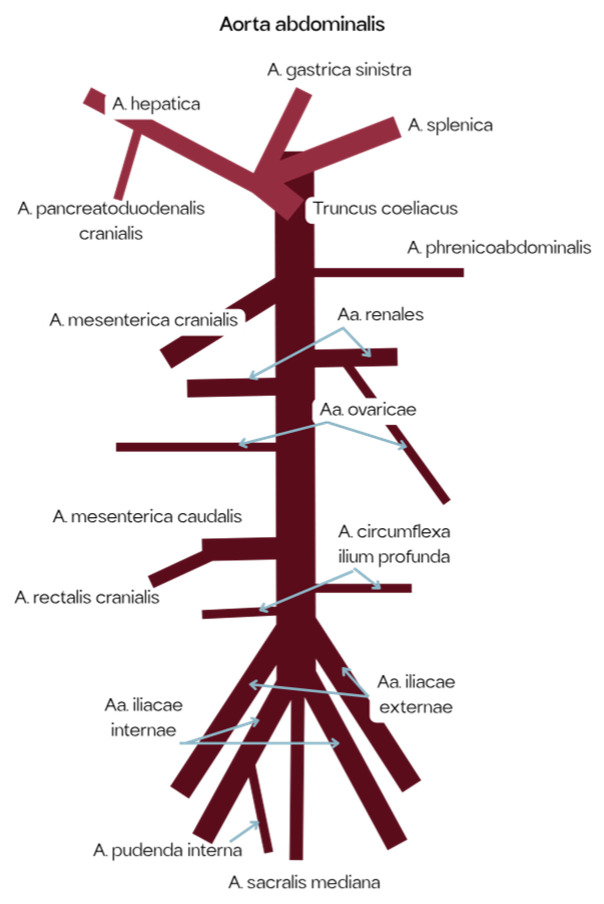
Schematic representation of the canine abdominal arterial system integrated into the simulator [14].

The branching pattern reconstructed in the model is illustrated in Figure 3, which shows the main arterial pathways and their connections as integrated into the simulator.

### 2.3. Pre-Training Assessments

Phase 1: Demographic data and baseline assessment

**Figure 4:**
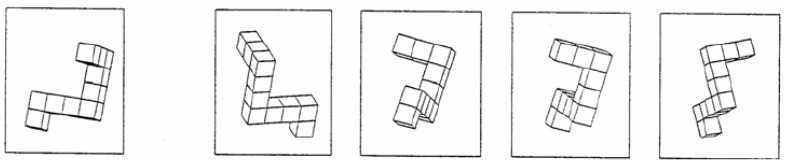
Example of MRT (Mental Rotation Test) [15].

At the start of the session, participants completed the Mental Rotation Test (MRT) (Figure 4) [15]. and filled out an initial questionnaire accessed through a QR code displayed in the room. The questionnaire collected general demographic information (sex, age, hobbies, work experience) and incorporated self-assessment questions (scored from 0 to 10, with 10 representing perfect knowledge) on both anatomical and interventional knowledge. Furthermore, participants were tasked with responding to theoretical questions of varying complexity in both domains, thus enabling a comparison to be made between their perceived knowledge and their actual level. Additionally, it was assessed whether participants played video games to evaluate the potential influence on 3D vision.

The MRT was used to evaluate spatial orientation skills. Participants were shown a target figure with four options and had to identify the two figures that matched the target afterward rotation. The shapes were block-based and presented in various orientations. As spatial orientation plays a crucial role in vascular interpretation and endovascular navigation, this test was included to examine whether individual differences in spatial ability influenced performance. The MRT was completed in two 3-min blocks separated by a 4-min interval, during which the demographic questionnaire was completed by the participants.

Phase 2: Simulator-based practical tests

**Figure 5:**
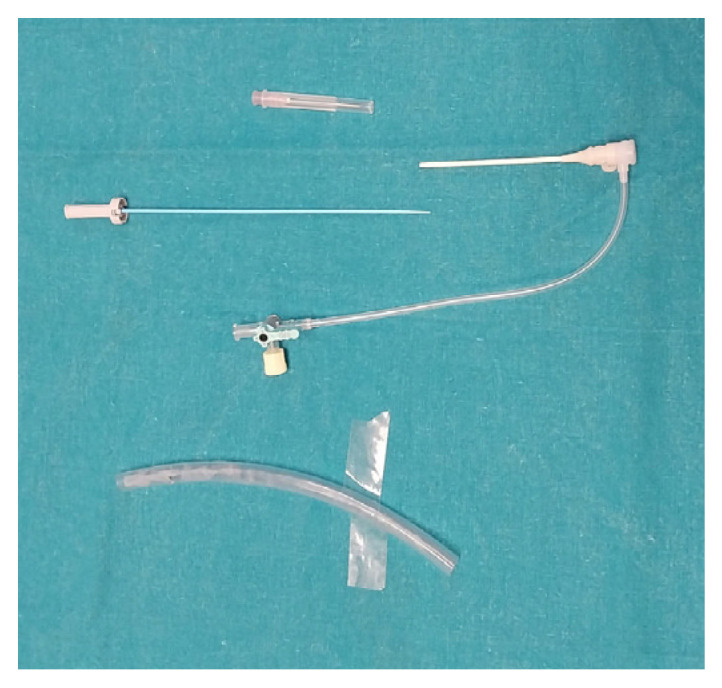
Mock vessel and 7 Fr vascular access.

**Figure 6:**
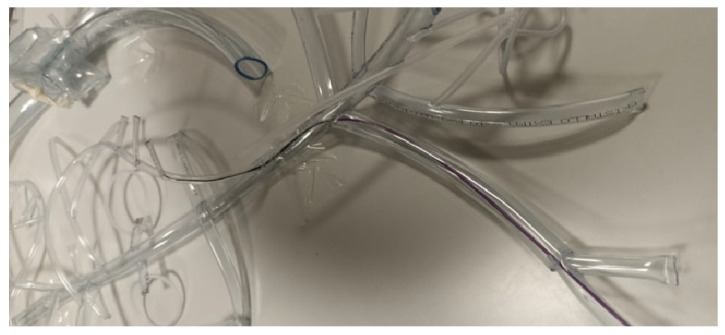
Endovascular catheterization test.

- Phase 2.1: Anatomical identification task: Participants were asked to identify the A. linealis and the A. mesenterica caudalis on Simulator 1. This test aimed to evaluate the participant’s understanding of vascular anatomy. Both accuracy and proximity to the correct location were scored.
- Phase 2.2: Seldinger technique: Participants had to perform the Seldinger technique using a mock vessel and a complete 7 Fr vascular access kit (introducer, dilator, guidewire, and puncture needle) (Figure 5), along with a scalpel for expanding the access site. Successful completion required the proper assembly and sequencing of the components. If participants were unfamiliar with the procedure, they were encouraged to attempt an intuitive assembly based on their interpretation of the instruments.
- Phase 2.3: Vascular catheterization: Participants were presented with a selection of catheters, guidewires, micro-guidewires, and micro-catheters and were asked to select the most appropriate option to reach the A. mesenterica cranialis using Simulator 2 (Figure 6). They were given a 5-min time limit. Outcomes assessed included material selection, navigation approach, arterial selectivity, accuracy of the final location, and total time used.

### 2.4. Training Session

An educational session followed the initial assessments. First, a guided anatomical review was conducted using Simulator 2, during which all vascular branches were labelled with sticky notes—except one, which was used as a control question in the final evaluation.

A concise explanation of interventional instruments was subsequently provided, encompassing their clinical indications and the optimal selection process. The Seldinger technique was then demonstrated, followed by a live demonstration of navigating the vascular system using the simulator and performing selective catheterization.

After this instruction, participants were given time to revise the content and practice freely with both simulators. They could attempt the vascular access technique again and revisit anatomical landmarks at their own pace. No time limit or direct supervision was imposed, although clarification was provided if requested.

### 2.5. Post-Training Assessments

The practical tests conducted after the instructional session mirrored those performed in the initial phase, maintaining the same structure and level of difficulty. This final evaluation phase consisted of the following tasks:

(1) Anatomical identification task: Participants were asked to identify the Truncus coeliacus, A. gastrica sinistra and A. rectalis cranialis on Simulator 1.

(2) Seldinger technique: Participants were required to repeat the vascular access procedure using the same mock setup as in the pre-training phase.

(3) Interventional navigation: Participants were instructed to navigate to the A. hepatica using Simulator 2, again within a 5-min time limit. As in the initial test, data were collected on material selection, navigation strategy, anatomical accuracy, and total time taken.

(4) Final questionnaire: A final questionnaire was completed via QR code, featuring new theoretical questions on vascular anatomy and interventional techniques. Participants also evaluated their perceived learning and the educational value of the simulator using a 5-point Likert scale.

### 2.6. Statistical Analysis

Description of qualitative variables were carried out with relative frequencies (%), while quantitative variables were described with mean, standard deviation (SD), minimum and maximum.

Association between two qualitative variables was assessed with Pearson’s Chi-square test (when less than 20% of expected values were lower than 5), using as alternatives Fisher’s exact test (for 2 × 2 contingency tables) or likelihood ratio test (for other tables). For paired qualitative variables, we used the McNemar’s test and for paired ordinal variables we apply the Somers’ D test.

Association between a quantitative and a binary qualitative variable was assessed depending on normality of quantitative variable (checked with Shapiro–Wilk test). Student’s *t* test for independent samples was selected as parametric test for comparison of two independent categories, and Mann–Whitney U test as non-parametric test. For comparison of paired samples, Student’s *t* test for paired samples was selected as parametric test, and Wilcoxon test as non-parametric test.

Statistical analysis was performed with IBM SPSS 30.0 for Windows, and error alpha was set at 0.050.

## 3. Results

### 3.1. Demographic Characteristics of the Study Population

A total of 80 individuals participated in the simulation sessions. Of these, 25% were staff from the Faculty of Veterinary Medicine, and 10% were residents at the University Veterinary Hospital. Both groups were classified as “graduates” for the study. The rest were undergraduate veterinary students, referred to throughout as “students”.

No significant gender differences were observed between the groups. However, there was a statistically significant age difference, with the “graduates” group being notably older (Table 1).

The overall percentage of participants with surgical theater experience was 37.5%. Among them, the majority (63.3%) had less than one year of experience, and only 13.5% had more than 5 years of surgical experience. However, 80.3% of participants reported having heard of vascular interventional procedures; of these, 20.9% were unable to name any specific technique, while 31.3% mentioned stent placement as the most familiar procedure.

A total of 60% of participants (48/80) stated they did not actively play video games. The group with the highest number of gamers was the student group, with 23 individuals. The Pearson’s Chi-square test (*p* = 0.293) revealed no statistically significant differences between the groups. Nevertheless, significant differences were observed concerning academic level and gender (*p* = 0.045 and *p* = 0.032, respectively), showing that male participants reported gaming at twice the rate of their female counterparts.

MRT

The results of the MRT (Table 2) showed no significant differences in scores between groups (*p* = 0.190) or between sexes (*p* = 0.289). When stratifying by sex within each group, no significant differences were found between graduates and students either (*p* = 0.274 in both cases), which may be attributed to the heterogeneity of the data and the relatively small sample size.

**Figure 7:**
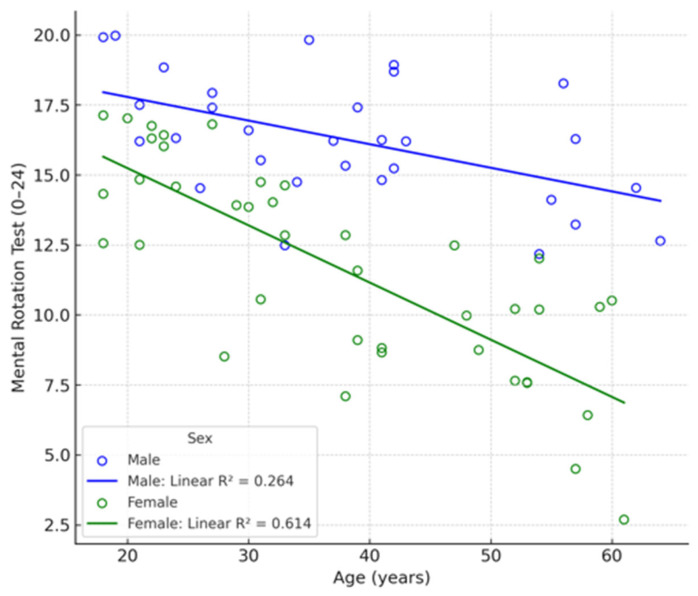
Distribution of MRT scores by gender and age. Significance according to the Mann–Whitney U test: *p* = 0.190.

However, when stratifying by both sex and group, a clearer downward trend in MRT scores with increasing age was observed in the female subgroup of the “graduates” group, as shown in Figure 7.

### 3.2. Theoretical Questionnaire Results

The training session significantly improved participants’ perceived learning in both areas, as indicated by the results of the self-assessment questionnaires on knowledge of interventional procedures and abdominal vascular anatomy (Table 3). Regarding the question about vascular access using the Seldinger technique, only 31.3% of participants were able to name it correctly before the session. In contrast, in the final questionnaire, 93.8% answered correctly.

Among those who had answered incorrectly on the first attempt, 90.9% provided the correct answer in the post-session test, while all participants who had answered correctly initially maintained their correct response. The findings indicated a statistically significant enhancement among the subjects who initially demonstrated an inability to identify the technique (*p* < 0.001, McNemar’s test).

However, with regard to the questions on vascular catheters, although there was an increase in the number of correct responses after the training, the difference was not statistically significant (*p* = 0.943) (Table 4).

The questions assessing knowledge of abdominal vascular anatomy showed an increase in the number of correct answers across all items, although the improvement was not statistically significant in every case. The lack of significant difference observed for certain vessels, such as the A. rectalisis cranialis (Table 5), was expected because this artery was not specifically addressed during the training session and therefore served as an internal control for the assessment.

A correlation analysis between the self-perception of anatomical knowledge and the actual results in the pre-session abdominal vascular anatomy questions showed no proportional relationship (Somers’ D = −0.022, *p* = 0.816). Participants exhibited a tendency to assign themselves higher scores than their demonstrated knowledge would justify. However, in the post-session analysis of the same variables, a positive correlation was observed between self-assessed knowledge and actual performance (Somers’ D = 0.317, *p* < 0.001).

The greatest increase in self-assessed knowledge was observed in the student group, although both students and graduates showed significant improvements in their perceived knowledge after the training session, as confirmed by the likelihood ratio test (*p* < 0.001). In the student group, over 30% of participants reported a 5-point increase in their perceived knowledge level. This improvement was directly correlated with the anatomy test results, with 53.8% (28/52) students showing an improvement in their scores, while 28.8% (15/52) maintained the same level.

In the graduate group, improvement was recorded in 53.6% (15/28) participants. The differences in self-perceived knowledge scores between the groups were attributed to the fact that graduates started with a higher baseline.

Finally, the theoretical question regarding the vascular route from the femoral artery to the liver demonstrated a significant improvement, with correct answers increasing from 43.8% (35/80) before the session to 82.5% (66/80) afterwards. This change was statistically significant (*p* < 0.001).

### 3.3. Practical Test Results: Anatomical Identification, Vascular Navigation and Access

**Figure 8:**
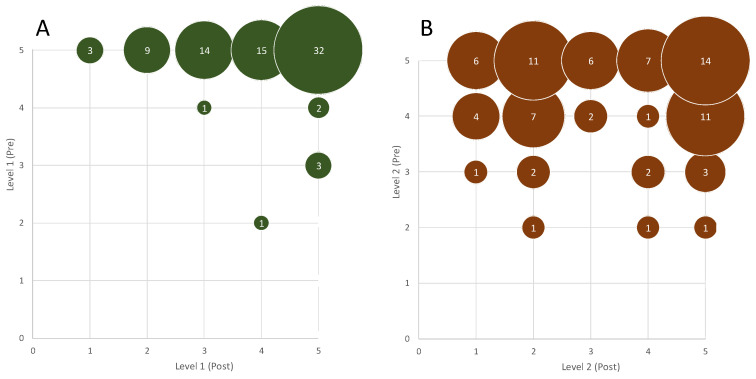
Bubble chart of Level 1 (**A**) and Level 2 (**B**) categorized by response accuracy (Anatomical Proximity, scale 1–5) comparing pre- and post-Intervention Performance: Bubble chart illustrating the accuracy of artery localization, with pre-intervention values on the X-axis and post-intervention values on the Y-axis. Each bubble represents an individual measurement, categorized according to anatomical proximity (scale 1–5). Bubble size reflects sample weight. Data highlight the overall improvement in accuracy after the intervention compared to baseline.

The initial practical tests involving the identification of vascular structures on the simulator mounted on the skeletal model are illustrated in bubble charts (Figure 8A), which shows the distribution of responses according to their proximity to the correct anatomical location. In the level 1 identification task, 46.3% of participants correctly identified the target artery. In the post-training level 1 test, this percentage increased to 91.3%, demonstrating a substantial improvement in anatomical localization. This remarkable progress can be attributed to the initial heterogeneity in participants’ anatomical knowledge and, more specifically, to the fact that many of them had never visualized abdominal anatomy as a three-dimensional tubular system. After the guided training session, the majority were able to clearly conceptualize the vascular network and its spatial relationships, which greatly facilitated accurate identification.

The more challenging identification task (level 2) did not show a statistically significant performance improvement (Figure 8B). It is hypothesized that this may be because the target artery in the post-session test was not labelled during the explanatory phase (*p* = 0.085). The percentage of correct responses rose from 36.3% to 55.0% between the pre- and post-training assessments.

An analysis was conducted to determine whether participants with better visuospatial ability (as indicated by higher MRT scores) were more accurate in identifying arteries on the skeletal model after the training session. Before the session, no significant differences were observed. However, following the anatomical explanation on the supine-mounted simulator, a statistically significant difference was detected between MRT performance and correct identification, according to the Kruskal–Wallis test (*p* = 0.017).

The second practical task involved catheterizing the designated artery, a procedure which required anatomical knowledge, appropriate selection of interventional materials, and navigation ability (Table 6). Before the training session, 48.8% (39/80) of participants held the conviction that no guidewire was required, while 33.8% (27/80) selected a hydrophilic guidewire based on intuition. Following the session, 98.8% of participants utilized a guidewire, and in the majority of cases, this was the correct implement, although this enhancement was not statistically significant (*p* = 0.694). Concerning the selection of catheter, it was considered that the use of a diagnostic catheter (e.g., a pig-tail) was the only incorrect procedure. This explains the high accuracy rate from the outset.

Only 10.0% of participants were able to successfully perform vascular navigation in the initial test; in contrast, 91.3% completed the task correctly in the final assessment, demonstrating a significant learning outcome (*p* < 0.001). A similar result was observed for successful vessel selectivity: only 6.3% of participants achieved this without prior instruction, whereas 87.5% did so following the training session (*p* < 0.001).

The average time required by participants who correctly reached the target artery is summarized in Table 7. A statistically significant difference in completion time (*p* = 0.022) was observed only among participants who successfully completed the navigation in both the initial and final tests. Initially, 38 participants correctly identified the destination artery, and 68.4% completed the task in less than five minutes. After the training session, all participants correctly identified the destination artery, and 64 completed the task within the same time limit. These results show a clear increase in the number of participants who achieved complete navigation after the training exercise.

The progression of learning was also assessed by comparing the performance levels of the subjects both prior to and following the training, in terms of both target identification and successful navigation. In the final test, 80.0% of participants (64/80) completed the task correctly. As illustrated in Table 8) a decline in performance was observed in only two individuals in comparison with their initial test results.

When segmenting participants who correctly identified the target artery, a statistically significant difference was observed in the time required to complete the task (*p* = 0.022). Initially, 38 participants correctly identified the destination, but only 68.4% did so within the 5-min time limit. In the post-training test, all participants correctly identified the destination, although only 64 completed the navigation within the allotted time.

Learning progression was further analyzed by distinguishing participants who correctly identified the vascular destination and navigated appropriately in the final test. A total of 80% (64/80) achieved this outcome, and only two participants showed a decline in performance compared to their initial results (Table 8).

The final practical test involved the execution of the Seldinger technique. In the pre-training phase, only 9 participants (11.3%) performed the procedure correctly, while 29 (36.3%) managed to partially assemble the access system. Following the instructional session, 93.8% of participants successfully executed both the assembly and procedural steps. The residual errors observed in the final test were predominantly exhibited by individuals who had previously demonstrated an inadequate performance of the technique during the preliminary phase.

### 3.4. Final Evaluation of the Experience

In the closing questionnaire, participants were asked to evaluate the overall session. All respondents (100%) considered its inclusion in the veterinary curriculum highly appropriate, and 78 out of 80 classified it as essential.

## 4. Discussion

Simulation-based education has become a cornerstone in the evolution of health professions training. As highlighted by Okuda et al. (2009), simulation offers a safe, standardized, and repeatable environment in which learners can actively develop both procedural and cognitive skills without exposing patients to risk. This pedagogical approach supports deliberate practice, immediate feedback, and the integration of theory into hands-on application-key pillars of adult learning theory and competency-based education [3,16]. In this context, the present study evaluated the effectiveness of two anatomical simulators representing the canine abdominal arterial system and their educational impact on veterinary students and graduates.

This study demonstrated that the use of the canine vascular simulators led to significant improvements in both theoretical knowledge and practical skills related to vascular anatomy and interventional techniques. Notably, participants showed measurable gains in identifying anatomical structures and understanding arterial distribution, as reflected in their post-session test scores. These results are in line with previous studies, which reported improved anatomical retention through physical models [6,16], and that simulation-based teaching enhances theoretical knowledge in guided interventions [2].

The improvement was even more pronounced in practical tasks, especially in the execution of the Seldinger technique and endovascular navigation. In the preliminary evaluations, a significant proportion of participants encountered difficulties in assembling the access kit or followed erroneous steps. In the post-training evaluation, the majority of participants demonstrated a reduction in the time taken to complete the technique, along with improved sequencing accuracy. This finding is consistent with the observations reported by Hunt et al. (2022), who noted that simulation not only improves technical competency but also increases confidence and autonomy among learners [5]. Similarly, Guimbarda et al. (2023) demonstrated that hands-on simulation courses significantly enhance procedural comfort among human radiology residents, particularly in ultrasound-guided vascular access using the Seldinger technique [13]. Their findings support the idea that structured simulation experiences can bridge critical educational gaps, even at advanced stages of medical training, further validating the approach used in our veterinary education setting.

The vascular navigation test also demonstrated a clear learning effect, with success rates increasing from less than 50% before the session to over 85% after training (*p* = 0.022). This significant difference was observed only among participants who successfully completed the navigation in both tests. Therefore, the improvement was mainly related to a higher number of participants achieving complete and accurate catheterization rather than to shorter navigation times. These findings suggest that the training enhanced procedural understanding, spatial orientation, and confidence during endovascular navigation. Such progressive improvement is consistent with the findings of Lioce et al. (2015) and Setin et al. (2018), who emphasized the role of simulation in supporting skill acquisition through repeated, structured practice [17,18]. These findings are especially relevant when considering the broader educational gap in interventional radiology. In the field of human medicine, where IR has already been firmly established, trainees continue to report a paucity of opportunities to practice vascular procedures [2,12,19,20]. According to Makris et al. (2020), a significant number of residents believe that early exposure to IR would have influenced their career trajectory. However, most receive little to no hands-on training during their foundational years [11]. This challenge is even more pronounced in the domain of veterinary medicine, where IR is noticeably absent from the core curricula. In this light, the use of a vascular simulator represents not only an effective learning tool but also a critical gateway to introduce students to a specialty that is frequently overlooked until advanced stages of training [9,21]. Encouraging early engagement may be key to cultivating a new generation of veterinary professionals interested in developing the field further.

Participants also reported enhanced self-perception of their knowledge in vascular anatomy and interventional radiology. The final questionnaire reflected a marked increase in their self-rated competence, which is known to positively influence learner engagement and performance. As Scalese and Issenberg (2005) observed, emotional reinforcement through confidence-building is a crucial factor in both academic and clinical preparedness [1].

The analysis of individual variables indicated that younger participants demonstrated superior overall performance, particularly in practical tasks. This phenomenon may be attributed to increased exposure to digital tools or visual-spatial activities. Conversely, participants with higher MRT scores exhibited superior anatomical localization, thereby substantiating the significance of spatial cognition in endovascular navigation. These results are consistent with those of Braid (2022) and Setin et al. (2018), who found a correlation between visuospatial ability and improved performance in simulation-based tasks [6,17].

Although not decisive, prior video game experience also appeared to positively influence task execution, especially in aspects requiring precision, hand-eye coordination, and time-sensitive decisions. This association has been previously documented in medical and veterinary contexts, where video game use has been shown to enhance fine motor skills and rapid decision-making [5,21].

One notable design element of this study was the intentional use of two different simulators across the training and evaluation phases. Instruction was delivered using Simulator 2 (supine model with visual organ references), while the final assessments were performed using Simulator 1 (skeleton-mounted in standing position). This setup allowed for the assessment of knowledge transfer across contexts—testing not only memory recall but also spatial adaptability. This type of cross-simulation evaluation is rarely applied in veterinary education and enhances the value of the model presented here.

Students’ perception of the simulator was overwhelmingly positive. They cited its clarity, realism, and usefulness in reinforcing anatomy and practicing interventional procedures in a low-risk environment. These impressions align with previous research highlighting strong student engagement and satisfaction with simulation-based resources [4,6,20,21].

These findings highlight the educational value of simulation-based tools in veterinary training and support the integration of such models into structured learning environments to bridge the gap between theoretical knowledge and clinical application. However, it is important to acknowledge the limitations of this approach. The absence of a control group is a limitation of the study. Although two groups were included, both received the same instructional intervention, and neither served as a true control group. As a result, the pre–post comparisons cannot isolate the specific effect of the simulator from other possible influences, such as increased familiarity with the test format, the natural progression of learning during the session, or the guidance provided during the practical activity. For this reason, the improvements observed after training should be interpreted as short-term learning gains associated with the session, rather than as evidence that the simulator alone accounts for the magnitude of the change.

To partially mitigate this limitation, one of the arteries (A. rectalis cranialis) was intentionally omitted from the explanatory session. The decline in correct responses for this item in the final questionnaire provides indirect evidence that the improvements observed in the other anatomical items were linked to the guided training rather than to repeated testing. Furthermore, the evaluation’s short-term nature precluded the assessment of long-term knowledge retention, and the utilization of a single evaluator hindered the analysis of inter-rater reliability. Another limitation of this study is the lack of formal validation of the simulator. Future research should include objective validation methods to confirm the anatomical accuracy and reproducibility of the model. Comparative analyses between the simulator’s vascular geometry—particularly branch angles and arterial origins—and canine CT angiograms could help establish its anatomical fidelity and strengthen its educational value.

Beyond navigation skills, future studies should also explore the potential of this simulator for evaluating the deployment and behavior of endovascular devices. The model could provide a reproducible and low-cost platform to assess the mechanical performance and delivery precision of catheters, guidewires, and stents before animal or preclinical trials. Such applications would help refine device design and operator technique in a controlled setting, consistent with recent advances in preclinical testing of absorbable metal stents [22,23]

It is recommended that future studies address these aspects and explore broader curricular integration across diverse veterinary education settings.

## 5. Conclusions

This study confirms the educational value of simulation-based tools in addressing one of the most challenging areas of veterinary training: the integration of anatomical knowledge with procedural skill development. The use of a handcrafted, low-cost canine vascular simulator allowed students and recent graduates to improve their understanding of abdominal vascular anatomy and to gain hands-on experience with interventional techniques such as vascular catheterization and the Seldinger procedure.

By combining theoretical evaluation, manual tests, and self-assessment, the study demonstrated significant learning gains and high user satisfaction. Importantly, the simulator served not only as a training resource but also as a strategic entry point to introduce students to the field of interventional radiology—an emerging specialty still underrepresented in the syllabus of Veterinary Bachelor.

The simplicity, affordability, and adaptability of the proposed model make it a promising option for broader educational use, especially in institutions with limited access to advanced simulation technologies. Its implementation may contribute to earlier and more equitable exposure to specialized clinical procedures, ultimately supporting the development of a new generation of veterinary professionals equipped with essential technical and spatial competencies. Although the study design does not allow causal inferences, the results support the value of incorporating low-cost simulators as complementary tools within structured veterinary training programs.

## Figures and Tables

**Table 1:** Demographic characteristics of population.

Group	n	Gender		*p*	Age				*p*
		Man	Woman		Media	DE	Min	Max	
Student	52	21.2%	78.8%	0.083 ^a^	23.25	3.803	19	39	<0.001 ^b^
Graduate	28	39.3%	60.7%		41.75	12.958	23	64	
Total	80	27.5%	72.5%						

^a^ Significance based on the Pearson’s Chi-square test. ^b^ Significance based on the Mann–Whitney U test.

**Table 2:** Mental Rotation Test Results per Group (MRT: 0–24).

Group	Media	DE	Min	Max	*p*
Students	10.37	5.280	4	22	0.190
Graduates	8.36	4.084	2	16	

Significance groups based on the Mann–Whitney U test.

**Table 3:** Self-perception of knowledge before and after the training session.

Item	Self-Assessment of Knowledge					
	n	Mean	SD	Min	Max	*p*-Value
IR pre	80	1.68	2.04	0	8	<0.001
IR post	80	5.25	2.16	1	10	
Anatomy pre	80	2.65	1.81	0	8	<0.001
Anatomy post	80	5.79	2.14	1	10	

Significance according to Wilcoxon Test. IR pre: questions regarding interventional radiology previous to the explicative session. IR post: questions regarding interventional radiology after the explicative session.

**Table 4:** Categorized Contingency Table of Interventional Radiology Test.

Test		Vascular Access Technique (Post)		
		Incorrect	Correct	*p*-Value
Vascular access technique (pre)	Incorrect	5 (9.1%)	50 (90.9%)	<0.001 ^a^
	Correct	0 (0.0%)	25 (100.0%)	
	Total	5 (6.3%)	75 (93.8%)	
		Catheter choice (post)		
		Incorrect	Correct	*p*-value
Catheter selection (pre)	Incorrect	29 (54.7%)	24 (45.3%)	0.943 ^b^
	Correct	15 (55.6%)	12 (44.4%)	
	Total	44 (55.0%)	35 (45.0%)	

^a^ Significance based on McNemar’s Test. ^b^ Significance based on Mann–Whitney U test.

**Table 5:** Theoretical test on anatomical knowledge.

Test	Correct Answer (Pre)	Correct Answer (Post)	*p*-Value
Spleen vascularization	64 (80.0%)	75 (93.8%)	0.003
A. rectalisis cranialis origin	71 (88.8%)	50 (62.5%)	0.785
Femoral artery origin	43 (53.8%)	69 (86.3%)	0.001

Significance based on McNemar’s Test.

**Table 6:** Correct vs. incorrect selection of interventional materials.

Material	n	Initial Test		Final Test		*p*-Value
		Correct	Incorrect	Correct	Incorrect	
Guidewire	80	33.8%	66.2%	98.8%	1.3%	0.694
Catheter	80	86.3%	13.8%	100.0%	0.0%	-

Significance based on the likelihood ratio test.

**Table 7:** Time (min) spent on accepted navigation tasks (only times < 5 min).

Test	n	Mean	SD	Min	Max
Initial experience	24	02:56	00:58	00:40	05:00
Final experience	64	02:59	00:54	01:12	04:59

**Table 8:** Comparison of initial and final navigation test results.

Identify the Target Artery (Pre)	Successful Completion of Pre-Training Navigation Test	n	Access to the Target Vessel (Post-Training)		
			Correct Selectivity	No Selectivity Achieved	*p*-Value
Failure to identify the target artery (n = 42)	Random vessel selectivity	18	66.7%	33.3%	0.035
	No selectivity achieved	24	70.8%	29.7%	
Correct arterial localization (n = 38)	Correct selectivity	26	92.3%	7.7%	0.022
	No selectivity achieved	12	91.7%	8.3%	

Significance based on McNemar’s test.

## Data Availability

The original contributions presented in this study are included in the article. Further inquiries can be directed to the corresponding author.

## Abbreviations

The following abbreviations are used in this manuscript:

IR	Interventional Radiology
MRT	Mental Rotation Test
Fr	French
